# Prevalence, one week incidence and knowledge on causes of diarrhea: household survey of under-fives and adults in Mkuranga district, Tanzania

**DOI:** 10.1186/1471-2458-14-985

**Published:** 2014-09-22

**Authors:** Kijakazi O Mashoto, Hamisi M Malebo, Emil Msisiri, Emanuel Peter

**Affiliations:** National Institute for Medical Research, P.O. Box 9653, Dar es Salaam, Tanzania

**Keywords:** Diarrhea, Water, Hygiene, Sanitation

## Abstract

**Background:**

Diarrhea is known to be the major cause of mortality in children aged less than five years old. Although mortality from diarrheal disease is decreasing globally, morbidity is not. The objectives of this study were to determine the prevalence of diarrhea among under-fives and assess knowledge on causes of diarrhea among adults in Mkuranga district Tanzania.

**Methods:**

Interviews with heads of households and observations were the methods of data collection employed by this study.

**Results:**

The prevalence of diarrhea in children below the age of five years as reported by heads of households was 6.1% and most affected were children in age groups 12 – 17 and 18 – 23 months (11.6% and 15.8% respectively; p – value 0.001). The rate of diarrhea incidence was 1 episode per 10 children per week. The mean duration of diarrhea illness was 1.7 days. Most under-fives had diarrhea for one (38.1%) or two (24%) days. Respondents in the 4th least poor quintile were more likely to have comprehensive knowledge on causes of diarrhea compared to respondents in the 1st poorest quintile. Male respondents were two times more likely to have comprehensive knowledge than female respondents. Respondents with comprehensive knowledge on causes of diarrhea were less likely to have poor hand-washing practice and more likely to have received water, hygiene and sanitation education. Under-fives in age group 12 – 17 months and those from households with reported poor hand washing practice were more likely to experience diarrhea episodes.

**Conclusion:**

Although prevalence of diarrhea reported in this study is low, the one week incidence is moderately high but less severe. Majority of household respondents had inadequate knowledge on causes of diarrhea and poor hand-washing practice. There is a need to provide WASH education to improve their knowledge on causes of diarrhea and hand washing practice.

## Background

The incidence of diarrhea does not differ substantially between regions, but incidence and case-fatality ratios are much higher in low-income than in middle-income and high-income countries. The most important causes of death in children younger than 5 years are infectious diseases, especially pneumonia, diarrhea, and malaria [1]. Globally, in 2010, there were 1.73 billion cases of diarrheal disease and 2% of episodes progressed to severe disease [2]. Of 7.6 million deaths in children younger than 5 years in 2010, 64% (4.879 million) were attributable to infectious causes and 10.5% were caused by diarrhea [3]. The highest numbers of childhood deaths are in Sub-Saharan Africa where 50% of deaths are from diarrhea [2]. Diarrhea deaths in all children younger than 5 years decreased from 1.160 to 0.801 million, a 4% decrease in the mortality rate per year during 2000 – 2010 periods [3]. Although child mortality has been declining worldwide as a result of socioeconomic development and implementation of child survival interventions, the situation in Africa is still frightening. The average child in developing countries experiences three or more episodes of diarrheal disease each year, accounting for up to 4 billion cases annually [1].

An estimated 884 million people worldwide lack access to improved water sources [4]. Lack of access to safe drinking water, together with inadequate sanitation and hygiene, has been identified to be the main contributor to diarrhea infection and deaths globally [5]. Where safe water infrastructure is lacking, especially in rural areas of developing countries, drinking contaminated water is an important cause of diarrhea [6].

Lack of access to basic water supply and sanitation is a major problem in both rural and urban Tanzania. Less than half of the rural population in Tanzania has access to safe drinking water [7] Access to clean and safe water in rural areas has declined since 2001 – from 46% to 40% in rural areas between 2000/2001 and 2007 [8].

Diarrhea was the fourth contributor of outpatient visit and the fifth cause of Mortality among children under the age of five years in the year 2009 in Tanzania [9]. A child gets about 5 episodes of diarrhea per year and the most frequently affected regions in the country are Shinyanga, Mara, Rukwa, Dodoma, Mbeya, Coast and Kigoma [10]. Mkuranga was among the top ten districts in Tanzania leading for diarrhea among under-five children despite improvement in water, hygiene and sanitation by AMREF since 2001. The most recently study on prevalence of diarrhea among under-five children was conducted in semi-urban wards of Mkuranga district and reported the prevalence of 32.7% [11]. However, there is scarce information on the prevalence of diarrhea among children under-five years, and information on knowledge on causes of diarrhea among community members in rural areas of Mkuranga district.

Thus objectives of this study were to determine the prevalence of diarrhea among under-fives and assess knowledge on causes of diarrhea among adults in Mkuranga district Tanzania.

## Methods

### Study site and sampling procedures

Mkuranga district is located at eastern part of Coast region, Tanzania. It has a population of 222,921 distributed in a total of 18 wards. Four wards are categorized as semi-urban and hence were excluded from the sampling frame. From a list of 14 rural wards in Mkuranga district, two wards namely Nyamato and Panzuo were randomly selected. Five villages were randomly selected from the list of villages in each ward. Thus a total of 10 villages participated in the project. The selected villages from Nyamato ward were: Nyatunduru, Mkiu, Mvuleni, Mkanoge, and Kilimahewa Kusini. Villages selected from Panzuo ward were: Kibuyuni, Mkruwili, Nyatanga, Mbulani and Kinzae. Each village executive officer was asked to provide a list of households with at least one child below the age of 5 years. Using the list provided by village executive officer, 60 households from each village were randomly selected.

### Study population and Sample size

A cross sectional study design was conducted between August and October 2013. A total of 600 households were selected for this study. Heads of households and under-fives were the study population.

### Data collection method and analysis

Interviews with heads of households and observations were the methods of data collection employed by this study.

One research assistant visited between 15 and 20 households and conducted interviews with heads of the households. In the event head of household was not present, any member of household who aged 18 years or above was interviewed. The data collection tools were pre-tested in Kisarawe 2-weeks prior to the survey. The questionnaire was designed to capture respondents’ knowledge on causes of diarrhea, water, hygiene and sanitation, and on information on occurrence of episodes of diarrhea among children below the age of five years.

Knowledge on causes of diarrhea was assessed by 7 items which had yes and no responses. The final score was obtained by summing the items and then dichotomized into less and comprehensive knowledge.

Water, hygiene and sanitation education score was obtained by summation of 5 items that asked if the respondent had or had not received education on construction of improved latrine, environmental cleanliness, treating drinking water, safe storage of drinking water and avoiding water recontamination.

The respondent was asked when he/she wash his/her hands. There were 9 items with yes and no responses. The total hygiene score was obtained by summation of these 9 items and then dichotomized into satisfactory and unsatisfactory hygiene.

Sanitation score was constructed by summation of four main observations around the household. These included presence or absence of feaces around the homestead, presence or absence of latrine, presence or absence of utensil drying rack, and whether the latrine and environmental cleanliness was satisfactory or not. Presence was coded 1 and absence 2. Originally, latrine and environmental cleanliness were scored in a 4 Likert scale. Using a checklist, data collector scored 1 if latrine and environmental cleanliness of the household and latrine was very satisfactory, and 4 if latrine and environmental cleanliness very unsatisfactory. After summation of latrine and environmental cleanliness, a dichotomized variable was constructed. Then all items for sanitation variable were summed and dichotomized into satisfactory coded 1 and unsatisfactory coded 2. Respondent was asked if the child (name of child) had diarrhea in the previous seven days. If yes respondent was asked how long the diarrhea lasted (number of days with diarrhea). In addition, respondent was asked on which day or days the child had diarrhea. This question was asked just to counter check the duration of the diarrhea. To get the information of number of episodes of diarrhea the child had, respondent was asked how many times on (the day) the child defecate loose/fluid stool? Same question was asked if the child had diarrhea for more than one day.

Wealth index was assessed as an indicative of socioeconomic status according to a standard approach in equity analysis [12]. Household assets such as house, toilet, land, radio, motorcycle, bicycle, car, phone, cow, pig, goat, sheep, donkey, chicken and duck were recorded as 1 “available and functioning” and 0 not available or available not functioning. Use of sources of energy for cooking i.e. electricity, kerosene oil, fire woods, gas and solar were recorded as 1 “yes” or 0 “no”. Assets and sources of energy for cooking were analyzed using principal components analysis (PCA). The first component resulting from the analysis was used to categorize households into four approximate quintiles of wealth ranging from the 1st poorest to the least poor 4th quintile.

The data was analyzed using SPSS version 17. Continuous variables were summarized into mean and standard deviation while categorical ones were summarized into proportions. Chi- square test and multi nominal logistic regression were applied to determine factors influencing knowledge of causes of diarrhea. The dependent variables were prevalence and knowledge on causes of diarrhea while socio-demographics, WASH education, hand washing practices and observed sanitation were independent variables. The level of statistical significance was set at p < 0.05.

### Diarrhea prevalence

Diarrhea was defined as the passage of three or more loose or liquid stools per day [13]. To minimize recall bias, Benjamini and his colleagues suggested that studies should measure caregiver reported illness with a 7 day recall [14]. We chose recording diarrhea during a 7-day period to avoid information bias in diarrhea prevalence. In most circumstances, applying a 7-day recall period using point prevalence data may be the preferred choice for measuring prevalence [15]. We calculated the prevalence of diarrhea as the percentage of children suffering from diarrhea assessed during a 7-day period.

### Ethical considerations

Ethical clearance to conduct the study was obtained from the Medical Research Coordinating Committee (MRCC) through National Institute for Medical Research (NIMR) Secretariat in Tanzania. Permission to undertake the study at the selected wards was obtained from the District and local government authorities. The study respondents were also asked for their consent and choose to participate voluntarily after getting clear message following the informed consent process.

## Results

### General sample characteristics

Survey was conducted in 599 households in Panzuo and Nyamato wards. Head of one household refused to participate in the study. In a household mean number of people were 6 and in total there were 3,197 individuals living the surveyed households. Of those, 849 were children below the age five years with mean age of 26.7 months (SD = 15.2).

Majority of the household respondents (90%) were married and peasants (83.6%) with mean age of 37.3 years (SD = 12.6). Almost half of the respondents 295 (49.2%) were also mothers/caretakers of under-fives. Over 60% of all respondents were literate.

### Prevalence of diarrhea among children below the age 5 years

Data for diarrhea among under-fives was collected from 818 children (96.3% response rate). Reasons for not having diarrhea data for the 31 children under the age of five include refusal (ten) and moving out of the study village (twenty one).

**Figure 1:**
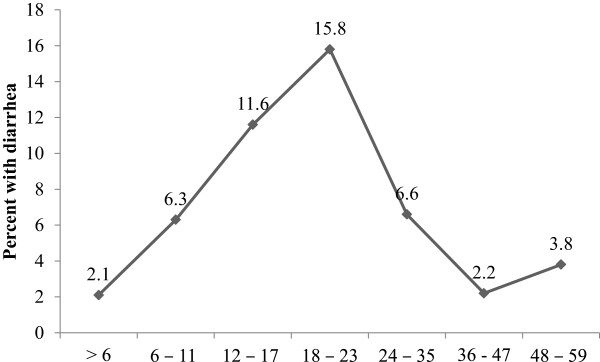
**Prevalence of diarrhea by age group in months.**

**Figure 2:**
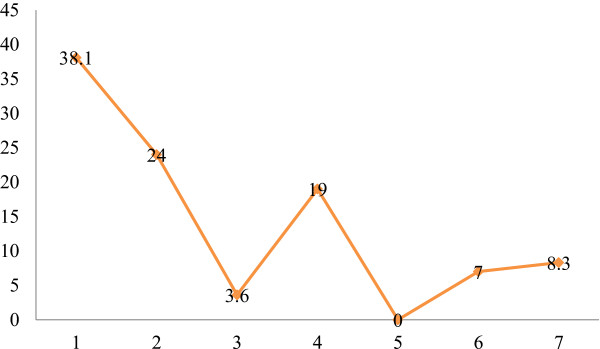
**Percentage distribution of under-fives by number of days with illness.**

The prevalence of diarrhea in children below the age of five years as reported by heads of households was 6.1%. Figure 1 depict the prevalence of diarrhea by age group. The prevalence of diarrhea was greatest (p < 0.001) in the age groups 12 – 17 and 18 – 23 months. The rate of diarrhea incidence was 1 episode per 10 children per week and over two-fifth of under-fives had diarrhea for 1.7 days (Table 1). Percentage distribution of under-fives by diarrhea duration is depicted in Figure 2.

**Table 1:** **Prevalence and incidence of diarrhea among under-fives by village (seven days recall period)**

Village	Reported children with diarrhea	Prevalence (%)	Diarrhea incidence	Observed children	Diarrhea incidence rate (episode per 10 children/week)	Diarrhea duration (days)
Kibudi	8	9.5	10	84	1.2	1.2
Kibuyuni	2	3.0	3	67	0.4	1.5
Kilimahewa	13	14.1*	32	92	3.5*	2.5*
Mbulani	1	1.3	0	77	0.0	0.0
Mkiu	4	4.9	4	82	0.5	1.0
Mkuruwili	6	8.3	8	72	1.1	1.3
Mvuleni	4	4.4	4	90	0.4	1.0
Nyamatotipo	5	5.7	9	88	1.0	1.8
Nyatanga	2	2.7	3	75	0.3	1.5
Nyatunduru	5	5.5	11	91	1.2	2.2
Total	50	6.1	84	818	1.0	1.7

*Chi-square test: p < 0.05.

### Reported hand washing practice

Although most of the respondents reported to wash their hands after using the toilet (82.3%) and before eating (91.5), use of communal pot 439 (73.3%), soap 534 (89.1%) and water 562 (93.8%) to wash hand is very common. The frequently mentioned reason for washing hand is to remove dirty (98.2%).

#### Knowledge on causes of diarrhea and water, sanitation and hygiene (WASH) education

A total of 306 respondents (51.7%) were not aware of the actual causes of diarrhea. Less than half of the respondents 285 (48.3%) had comprehensive knowledge on causes of diarrhea. Very few respondents knew that use of communal pot to wash hands may lead to transmission of microbes that cause diarrhea (Table 2). Nevertheless, only one third of the respondents reported to have some water, sanitation and hygiene (WASH) education. Kilimahewa village had significantly less number of respondents trained on WASH (Table 3). Association of knowledge on causes of diarrhea with wealth index, water, sanitation and hygiene is depicted in Table 4. High proportion of respondents with comprehensive knowledge on causes of diarrhea was in the 1st poorest quintile of the wealth index.

**Table 2:** **Number and percent (%) of respondents with correct knowledge on causes of diarrhea**

Drinking unsafe water	474 (79.9)
Eating contaminated fruits and vegetables	183 (31.0)
Not washing hands after using the toilet	296 (50.1)
Eating without washing hands	262 (44.3)
Using communal dish/pot to wash hands	112 (19.0)
Not using toilet	219 (37.1)
Bottle feeding using unclean bottle	84 (14.2)
Comprehensive knowledge score	285 (48.3)

**Table 3:** **Respondents with knowledge on diarrhea causes, water, sanitation and hygiene education, and good hand-washing practice by village**

Village	Respondents reporting good hand-washing practice N (%)	Respondents with comprehensive knowledge on diarrhea causes N (%)	Households with satisfactory sanitation N (%)	Respondents with some WASH education N (%)
Kibudi	29 (49.2)	35 (63.6)	47 (81.0)	12 (20.0)
Kibuyuni	19 (32.2)	16 (27.1)	54 (93.1)	14 (23.7)
Kilimahewa	42 (70.0)	46 (76.7)	52 (88.1)	10 (16.7)*
Mbulani	42 (70.0)	36 (60.0)	53 (88.3)	13 (21.7)
Mkiu	31 (51.7)	28 (46.7)	44 (75.9)	17 (28.3)
Mkuruwili	1 (1.7)*	15 (25.0)	32 (53.3)	24 (40.7)
Mvuleni	25 (41.7)	25 (41.7)	55 (91.7)	34 (56.7)
Nyamatotipo	28 (46.7)	10 (16.7)*	31 (51.7)*	14 (23.3)
Nyatanga	53 (88.3)	32 (54.2)	50 (83.3)	13 (21.7)
Nyatunduru	54 (90.0)	42 (73.7)	58 (96.7)	30 (50.0)
All	324 (54.2)	285 (48.3)	476 (80.3)	181 (30.3)

Chi-square test; *p < 0.001.

**Table 4:** **Association of knowledge on causes of diarrhea with wealth index, water, hygiene and sanitation (Those with comprehensive knowledge)**

Wealth index	Number (proportion expressed in %)	Odd ratio (95% CI)
1st poorest quintile	117 (58.8)**	1
2nd poor quintile	56 (44.8)	0.56 (0.36 – 0.89)
3rd poor quintile	54 (41.4)	0.49 (0.31 – 0.78)
4th least poor quintile	58 (42.6)	0.52 (0.33 – 0.81)
**Reported hand-washing practice**		
Poor practice	62 (22.7)	1
Good practice	223 (70.3)***	8.56 (5.76 – 12.7)
**Observed sanitation**		
Unsatisfactory	44 (37.4)	1
Satisfactory	241 (51.1)*	1.32 (0.81 – 2.15)
**Water, hygiene and sanitation education (WASH)**		
No education	170 (41.7)	1
Some education	115 (63.5)***	2.72 (1.78 – 4.15)

Chi-square test; *p < 0.05; **p < 0.001; ***p < 0.0001.

Respondents in the 1st poorest quintile were more likely to have comprehensive knowledge on causes of diarrhea compared to respondents in the 4th least poor quintile. Male respondents were two times more likely to have comprehensive knowledge than female respondents. Respondents with comprehensive knowledge on causes of diarrhea were less likely to have poor hand-washing practice and less likely to have never received water, hygiene and sanitation education (Table 5). Under-fives in age group 12 – 17 months and those from households with reported poor hand washing practice were more likely to experience diarrhea episodes (Table 6).

**Table 5:** **Multiple logistic regression of factors influencing knowledge on causes of diarrhea–respondents with comprehensive knowledge (R** ^**2**^ **= 0.445)**

Variable ^*^	OR (95% CI)
***Sex:***	
Male	1.99 (1.25 – 3.16)
Female	1
***Wealth index***	
1st poorest quintile	1.82 (1.08 – 3.06)
2nd poor quintile	1.07 (0.59 – 1.98)
3rd poor quintile	0.89 (0.51 – 1.58)
4th least poor quintile	1
***Reported hand-washing practice***	
Poor	0.11 (0.66 – 0.18)
Good	1
***Water, hygiene and sanitation education (WASH)***	
No education	0.30 (1.8 – 0.49)
Some education	1

*Controlled for literacy, number of under-fives in the household, villages and sanitation.

**Table 6:** **Multiple logistic regression of factors influencing prevalence of diarrhea in under-fives (these with diarrhea (R** ^**2**^ **= 0.171)**

Variable*	OR (95% CI)
***Child age groups in months***	
<6	0.66 (0.07 – 6.31)
6 – 11	2.07 (0.54 – 0.92)
12 – 17	4.09 (1.34 – 12.49)
18 – 23	3.51 (0.71 – 17.47)
24 – 35	2.26 (0.66 – 7.72)
36 – 47	0.38 (0.07 – 2.08)
48 – 59	1
***Village***	
Kibudi	0.35 (0.06 – 1.89)
Kibuyuni	0.75 (0.18 – 3.15)
Kilimahewa	1.17 (0.27 – 5.02)
Mbulani	0.19 (0.02 – 1.81)
Mkiu	0.34 (0.07 – 1.70)
Mkuruwili	0.21 (0.04 – 1.08)
Mvuleni	0.29 (0.06 – 1.52)
Nyatunduru	0.29 (0.04 – 2.06)
Nyatanga	0.86 (0.20 – 3.66)
Nyamatotipo	1
***Reported hand-washing practice***	
Poor	2.67 (1.01 – 6.55)
Good	1

*Controlled for literacy, number of under-fives in the household, child sex, respondent sex, sanitation, WASH education, Wealth index and respondents’ knowledge on causes of diarrhea.

## Discussion

Generally household respondents had inadequate knowledge on the causes of diarrhea. The knowledge of the household respondents had significant relation with their exposure to water, sanitation and hygiene education as well as the wealth quintile. This low level of knowledge had been noted in other studies in Ethiopia, Nigeria and Tanzania [11, 16–18]. The prevalence of diarrhea of 6.1% among under-fives in Mkuranga is similar to that reported in India [19] but is lower than that reported in the previous studies conducted in Tanzania and elsewhere [20–23]. The lower levels of reported prevalence of diarrhea among under-fives in this study could be due to the fact we interviewed heads of households and not mothers or care takers of the under-fives. The highest prevalence rates are reported for children aged 12 – 17 months, with the peak at 18 – 23 months. Similar trends have been reported in a study conducted in Ethiopia [24]. In that age group one in five children had diarrhea in the seven day period. After the second birthday, the prevalence of diarrhea declines rapidly with increasing age of the child. Similar trends have been reported in 1991 and 2005 in Tanzania [22, 23]. However, the TDHS of 2004–05 reports a diarrhea prevalence rate of 7 percent among infants less than six months of age which is higher than the prevalence among infants less than 6 months reported in the present study. The estimated prevalence of diarrhea among children below the age of five years in Mkuranga district, Coast region is similar to the prevalence of diarrhea in Coast region reported in 2010 Tanzania Demographic and Health survey [7] and in Burkina Faso [25].

Duration of diarrhea ranged between one and seven days. Over one third (38.1%) of under-fives had at least one day with diarrhea. Similar findings have been reported in under-fives from Pakistan [19]. Episode of diarrhea reported in this study is higher than the incidence reported in Mali, Mozambique, Kenya, India, Bangladeshi and Pakistani but similar to Gambia [19]. The fact that both male and female children were equally affected by diarrhea is in line with the findings of other studies [17]. Similar to what have been reported in a study conducted in Bangladeshi [26], wealth index did not significantly associate with the risk of diarrhea in under-fives. However, respondents in the 4th least poor quintile were more likely to have comprehensive knowledge on causes of diarrhea. Unlike the study conducted in Ethiopia [24], we did not find any association between diarrhea occurrence and number of under-fives in the household.

Contaminated hands are one of the most common modes for transmitting human pathogens [27]. Poor hand washing practices of the respondents associated with the occurrence of diarrhea. Under-fives from the household with reported poor hand washing practice were more likely to experience diarrhea. Unhygienic hand washing practice by using a communal pot was found to be rampant in the study areas as it was practiced by 73.3% of the visited households. Use of communal pot to wash hands increases the risk of diarrhea and it was fascinating to note that only 19% of the respondents knew this fact. Respondents with little knowledge on causes of diarrhea were more likely to have poor hand-washing practice.

Water, sanitation, and hygiene (WASH) improvements have the potential to reduce rates of diarrheal disease by preventing exposure to infectious pathogens. Respondents across all villages had less exposure to WASH education which might have contributed to the inadequate knowledge on causes of diarrhea. Male respondents and those with WASH education were more likely to have comprehensive knowledge on causes of diarrhea.

Validity of the presented results depends on the ability of the respondents to correctly ascertain diarrhea and recall episodes that occurred a week prior to survey. Furthermore, we interviewed heads of households who may not be able to recall if a child had diarrhea in the last seven days. Nevertheless, a study has shown that child caregivers fail to recognize half of diarrhea episodes that occur in their children [25]. Another limitation is that we report results of a single survey which did not account for seasonal differences in disease incidences. Therefore, the reported prevalence and incidence of diarrhea may be underestimated.

## Conclusion

This study reported low prevalence of diarrhea among children below the age of five years in Panzuo and Nyamato wards of Mkuranga district. The most affected age group being 12 – 17 months. Only one third of the respondents have ever received some WASH education and this is translated into inadequate knowledge on the causes of diarrhea and poor hand washing practice for most of the respondents. In turn reported poor hand washing practice of respondents increased the risk of diarrhea in under-fives. This indicates the need for provision of water, sanitation and hygiene education.
